# Targeted therapy acts to sensitize stereotactic body radiotherapy for pulmonary oligometastases from colorectal cancer

**DOI:** 10.3389/fonc.2025.1464707

**Published:** 2025-05-08

**Authors:** Xiye Sun, Pei Shu, Yali Shen, Zhiping Li, Ning Liu, Ganlu Ouyang, Yuanling Tang, Meijuan Huang, Xin Wang

**Affiliations:** ^1^ Division of Thoracic Tumor Multimodality Treatment and Department of Medical Oncology, Cancer Center, West China Hospital, Sichuan University, Chengdu, China; ^2^ Division of Abdominal Tumor Multimodality Treatment, Department of Radiation Oncology, State Key Laboratory of Biotherapy and Cancer Center, West China Hospital, Sichuan University, Chengdu, Sichuan, China; ^3^ Clinical Trial Center, National Medical Products Administration Key Laboratory for Clinical Research and Evaluation of Innovative Drugs, West China Hospital, Sichuan University, Chengdu, Sichuan, China

**Keywords:** stereotactic body radiotherapy, colorectal carcinoma, oligometastatic lung tumors, cetuximab, bevacizumab

## Abstract

**Background:**

Stereotactic body radiation therapy (SBRT) is used to manage lung metastases arising from colorectal cancer (CRC), but its effectiveness is constrained by the radioresistance of CRCs. Here, we explored whether concurrent therapy with cetuximab or bevacizumab could improve the prognosis of CRC patients with pulmonary oligometastases.

**Materials and methods:**

CRC patients with oligometastatic lung tumors (OLTs) treated with concurrent chemoradiotherapy from March 2011 to March 2023 were retrospectively analyzed. Treatment outcomes for local control rate (LCR), progression-free survival (PFS), overall survival (OS), and toxicities were assessed.

**Results:**

Sixty-nine patients were included, with a median follow-up of 34 months. The 1-year LCRs for SBRT + chemotherapy, SBRT + chemotherapy + bevacizumab, and SBRT + chemotherapy + cetuximab were 63.3%, 96.2%, and 94.4%, respectively. Incorporating bevacizumab or cetuximab significantly prolonged median OS compared to chemotherapy (61 vs. 46 vs. 24 months). Substantial differences in median PFS were noted, with durations of 5, 23, and 8 months for SBRT + chemotherapy, SBRT + chemotherapy + bevacizumab, and SBRT + chemotherapy + cetuximab, respectively. Our univariate analysis revealed that patients under targeted therapy of bevacizumab or cetuximab were linked to prolonged OS and PFS (*p* < 0.05). Tumor size <2 cm and median biologically effective dose (BED_10_) ≥100 Gy were correlated with higher local control rates (*p* < 0.05). Furthermore, comprehensive multivariate analysis confirmed that tumor sizes of <2 cm were linked to better local control (*p* < 0.05). All three combination regimens were well tolerated, and the occurrence of toxicities was higher in treatments involving targeted therapy.

**Conclusion:**

Combining concurrent chemoradiotherapy with cetuximab or bevacizumab improves treatment outcomes, with manageable toxicity. Given the limited sample size of this study, larger studies such as prospective trials are needed.

## Introduction

Colorectal cancer (CRC) ranks as the third main cause of death and the second leading cause of cancer-related deaths worldwide (1). The second most common site for metastases from CRC is the lung, detected in 10% to 20% of cases (2, 3). Surgery is the standard treatment for CRC patients with oligometastatic lung tumors (OLTs) (4). However, the median survival does not exceed 10 months without surgery, especially for patients where surgery is not an option (5–7). Stereotactic body radiation therapy (SBRT) has been reported to improve the local control of lung oligometastases from CRC, with a 1-year local control rate (LCR) ranging from 62% to 92% (8), which is suboptimal (9–11). Therefore, we and others in the field are exploring strategies to enhance the efficacy of SBRT. Our recent study demonstrated that an increased dose (60 Gy in 5 fractions) could lead to enhanced local recurrence-free survival in patients (12). However, limitations in normal tissue tolerance represent a confounder in achieving the desired radiation intensity during SBRT. Therefore, employing radiation sensitizers in conjunction with SBRT presents a viable strategic approach.

Prior research suggests that cetuximab and bevacizumab enhance radiosensitivity. In advanced head and neck cancer, combining cetuximab with radiotherapy significantly improved local control and survival (13, 14). Preclinical studies support this, showing that cetuximab enhances radiotherapy efficacy in CRC cells by inhibiting DNA repair (15, 16). Similarly, anti-Vascular Endothelial Growth Factor therapy has demonstrated radiosensitizing effects in preclinical models (17). In CRC patients with lung oligometastases, bevacizumab increased complete response rates by 21% following stereotactic ablative radiotherapy (18).

Currently, there is limited evidence for SBRT-based combination chemotherapy with targeted therapy including cetuximab or bevacizumab to manage CRC patients. Here, we reported a retrospective study to investigate whether bevacizumab or cetuximab could improve the radiotherapy sensitivity of CRC patients with OLTs.

## Materials and methods

### Patient characteristics

The CRC patients with OLTs who were treated with SBRT-based combination therapy in West China Hospital, Sichuan University, between March 2011 and March 2023, were evaluated. The selection criteria were as follows: age ≥ 18 years, the primary tumor was colorectal adenocarcinoma, the primary tumor had been surgically removed, OLTs arose postoperatively from CRC, distant metastasis to ≤2 organs (lung alone or lung combined with liver, lymph nodes, etc.), total metastasis number ≤5, and maximal tumor diameter ≤5 cm. The lung metastases were medically inoperable, and extra-lung tumor sites were controlled, with Eastern Cooperative Oncology Group performance score (ECOG-PS) ≤1. Response assessment adhered to the Response Evaluation Criteria in Solid Tumors (RECIST) 1.1 guidelines. CT scans were employed to appraise the irradiation target area, given that the challenges in discerning between recurrence and pseudoprogression following SBRT, the integration of FDG-PET, and the utilization of pathological biopsy, whenever feasible, contributed to a more nuanced and thorough evaluation. The pulmonary toxicity evaluation was based on Common Terminology Criteria for Adverse Events (CTCAE) 5.0 standards (19, 20).

### SBRT technique

All patients received SBRT for pulmonary oligometastases. For those whose respiratory movement (measured using IGRT motion view or Image-Guided Radiation Therapy with Four-Dimensional Cone-Beam Computed Tomography) was greater than 1 cm in the head–foot direction, active breath control (ABC) was used to control such movement. For respiratory movements less than 1 cm, four-dimensional CT (4DCT) was used to control respiratory movements. Gross tumor volume (GTV) was delineated using CT lung windows. The internal target volume (ITV) consisted of the union of 10 GTV contours of 4D breathing phases. The clinical target volume (CTV) corresponded to the ITV. The planning target volume (PTV) was defined as the ITV, the upper and lower sides were expanded by 10 mm, and the surrounding sides were expanded by 5 mm. Radiotherapy doses were administered depending on the size and location of the tumor. The radiotherapy dosage was determined based on the physician’s expertise, with the prescribed dose to the PTV ranging from 35 to 60 Gy in 3 to 10 fractions, three times per week. The PTV encompassed 80% of the isodose volume (ranging from 60% to 90%). To meet target dose criteria, more than 95% of the PTV must be covered, with 99% of the PTV receiving a prescribed radiation dose of at least 90% of the total. If the dose exceeds 105%, it should fall within the PTV range.

### Chemotherapy and targeted therapy

All the patients received concurrent chemoradiotherapy. Chemotherapy regimens included mFOLFOX6, FOLFIRI, and XELOX. During concurrent radiotherapy, chemotherapy was administered as planned, but the dose of chemotherapy could be properly adjusted based on the patient’s condition and tolerance (70%–80% of the usual dose could be administered). Before initiating cetuximab treatment, all patients underwent genetic testing. Those with RAS wild type could proceed with cetuximab with a dose of 500 mg/m^2^ every 2 weeks. The number of cetuximab administrations varied between two and four– times, depending on the radiotherapy dose group. The bevacizumab dose was 5 mg/kg every 2 weeks (mFOLFOX6/FOLFIRI-based regimens) or 7.5 mg/kg every 3 weeks (XELOX-based regimens). After synchronous treatment, maintenance therapy was tailored by the attending physician based on the patient’s condition.

### Follow-up

Efficacy evaluations were conducted 1 month after the end of radiotherapy and every 3–4 cycles during chemotherapy thereafter. After the treatment, follow-up and efficacy evaluations were typically conducted according to the requirements of the physician in charge. The evaluations were performed at least once every 3 to 6 months.

### Statistical analysis

Local control duration was measured from SBRT initiation to local progression. Overall survival (OS) duration was calculated from SBRT start to death. Progression-free survival (PFS) duration was calculated from the commencement of SBRT to tumor progression or death. LCR, PFS, and OS were all calculated using the Kaplan–Meier analyses. The results of subgroups were compared using the log-rank or Gehan–Breslow–Wilcoxon test. Univariate and multivariate Cox regression analyses were used to calculate the influence of variables on OS, PFS, and LCR. All statistical tests were two-sided. Values of *p*-value <0.05 were indicated as statistically significant. SPSS 20 (IBM Corporation, Armonk, NY, USA) and Prism 9 (GraphPad Software, San Diego, CA, USA) were used for statistical analyses.

## Results

### Patient characteristics

There were 86 lung oligometastases in 69 patients (41 men and 28 women) included. The median age was 61 (40–87). The number of lung oligometastases treated per patient ranged from one to three. Among the patients, 22 patients with OLTs originated from the colon, while others from the rectum. Before receiving SBRT, 27 patients had undergone treatments for liver metastases. Among them, 13 patients successfully underwent radical resection of liver metastases with no observed recurrence. Additionally, three patients underwent radiofrequency ablation, another three received local SBRT, and eight underwent systemic chemotherapy. All liver lesions were under stable control.

The treatment regimens administered were as follows: SBRT + chemotherapy for 25 patients, SBRT + chemotherapy + bevacizumab for 26 patients, and SBRT + chemotherapy + cetuximab for 18 patients. The median tumor size of OLTs was 1.4 cm (0.3–5.0 cm), and the median biologically effective dose (BED_10_) of SBRT was 105.6 Gy (range, 50.0–180.0 Gy). **Table 1** shows the characteristics of the patients. **Table 2** reveals the baseline characteristics of each patient group.

**Table 1:** Patients and tumor characteristics.

Baseline characteristics	All patients (n = 69)
Age (years)	
Median	61
Range	40–87
Gender, n (%)	
Male	41 (59.4%)
Female	28 (40.6%)
Primary site, n (%)	
Colon	22 (31.9%)
Rectum	47 (68.1%)
Previous site of metastases, n (%)	
Liver metastases	27 (39.1%)
Other sites (lymph nodes)	42 (60.9%)
Type of oligometastases	
Synchronous	6 (8.7%)
Metachronous	63 (91.3%)
Number of lesions per patient	
1 lesion	54 (78.3%)
2 lesions	13 (18.8%)
3 lesions	2 (2.9%)
Tumor size (cm)	
Median	1.4
Range	0.3–5.0
BED_10_ (Gy)	
Median	105.6
Range	50.0-180.0
Treatment, n (%)	
SBRT + chemotherapy	25 (36.2%)
SBRT + chemotherapy + bevacizumab	26 (37.7%)
SBRT + chemotherapy + cetuximab	18 (26.1%)

BED, biologically effective dose; SBRT, stereotactic body radiation therapy.

**Table 2:** Baseline characteristics of each patient group.

Characteristics	SBRT + chemotherapy	SBRT+ chemotherapy + bevacizumab	SBRT + chemotherapy+ cetuximab	*P*-value
Age				0.046
Median (range)	67 (42–87)	58 (47–75)	58 (40–76)	
M ± SD	65.0 ± 12.6	58.9 ± 7.6	57.9 ± 10.5	
Gender				0.46
Male	16	13	12	
Female	9	13	6	
Tumor size (cm)				0.45
Median (range)	1.5 (0.6-5.0)	1.4 (0.3–3.8)	1.2 (0.4-4.0)	
M ± SD	1.7 ± 1.2	1.4 ± 0.8	1.4 ± 0.9	
Primary site				0.43
Colon	10	6	6	
Rectum	15	19	12	
BED_10_ (Gy)				0.59
Median (range)	105.6 (72.0-132.0)	105.6 (60.0-132.0)	105.6 (50.0-180.0)	
M ± SD	106.9 ± 18.7	104.9 ± 23.6	112.4 ± 30.2	

BED, biologically effective dose; SBRT, stereotactic body radiation therapy.

### Local control

**Figure 1:**
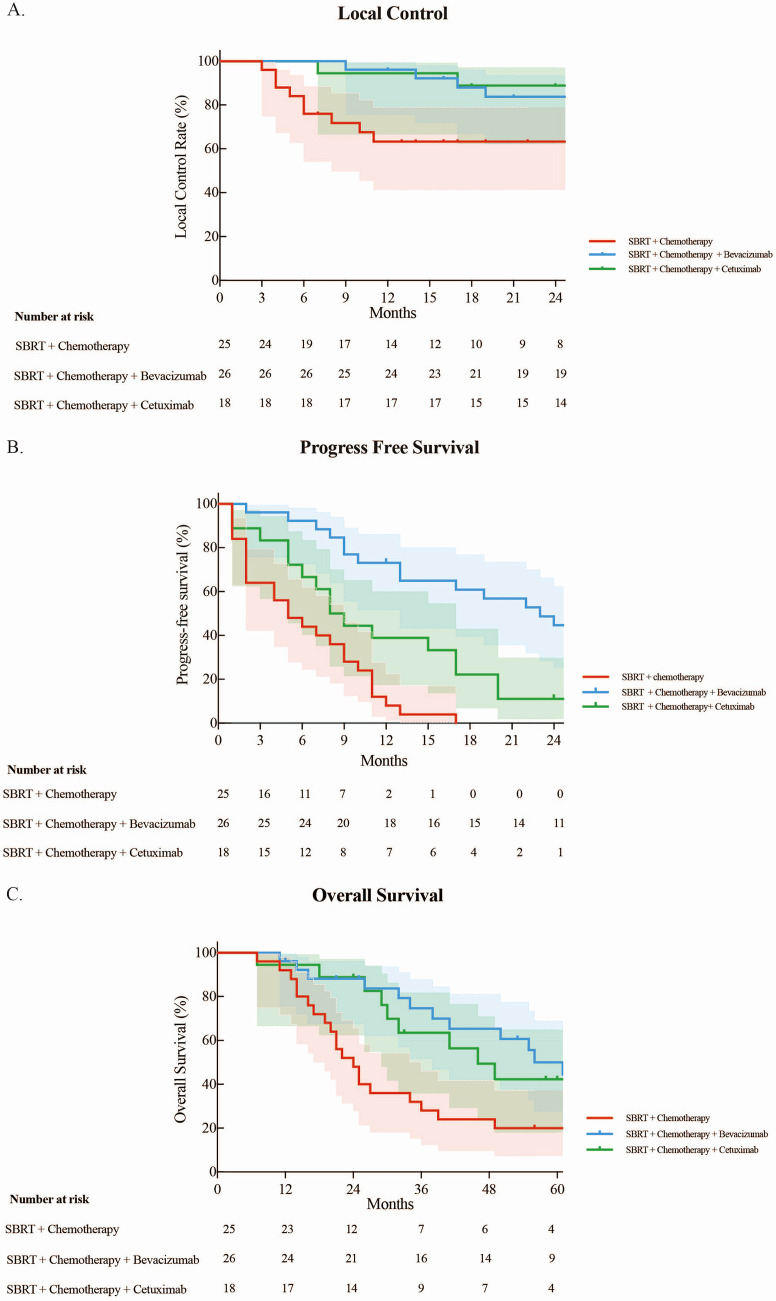
**(A)** Local control rate. **(B)** Progression-free survival. **(C)** Overall survival of three groups.

The median follow-up period after SBRT for OLTs from CRC was 34 months. The 1- and 2-year LCRs were 84.0% and 77.4%, respectively. Patients treated with bevacizumab or cetuximab had better local control (*p* < 0.05). The 1– and 2-year LCRs for the SBRT + chemotherapy arm, SBRT + chemotherapy + bevacizumab arm, and SBRT + chemotherapy + cetuximab arm were 63.3% vs. 96.2% vs. 94.4%, and 63.3% vs. 83.8% vs. 88.9%, respectively (**Figure 1A**). The 1- and 2-year LCRs for patients with one, two, and three lung lesions treated simultaneously were 81.4% vs. 92.3% vs. 50.0%, and 74.8% vs. 84.6% vs. 50.0%, respectively (*p* > 0.05).

### Progression-free survival

The median PFS for all included patients was 10 months, with the 1-, 3-, and 5-year PFS rates being 40.6%, 14.7%, and 10.7%, respectively. The median PFS rates for the SBRT + chemotherapy arm, SBRT + chemotherapy + bevacizumab arm, and SBRT + chemotherapy + cetuximab arm were 5, 23, and 8 months, respectively. Patients treated with targeted therapy had longer PFS compared with those treated with chemotherapy (*p* < 0.05). The 1-year PFS rates were 8.0%, 73.1%, and 38.9% for the SBRT + chemotherapy arm, SBRT + chemotherapy + bevacizumab arm, and SBRT + chemotherapy + cetuximab arm, respectively. All SBRT + chemotherapy patients experienced progression within 2 years. The 2-year PFS rates were 44.7% and 11.1%, respectively, in the SBRT + chemotherapy + bevacizumab arm and SBRT + chemotherapy + cetuximab arm (**Figure 1B**).

### Overall survival

The median OS for all 69 patients was 41 months with the 1-, 3-, and 5-year OS rates of 94.2%, 54.1%, and 37.0%, respectively. The median OS rates for the SBRT + chemotherapy arm, SBRT + chemotherapy + bevacizumab arm, and SBRT + chemotherapy + cetuximab arm were 24, 61, and 46 months, respectively. Patients treated with targeted drugs had longer survival compared with chemotherapy (*p* < 0.05). The 1-, 3-, and 5-year OS rates for the arms receiving SBRT + chemotherapy, SBRT + chemotherapy + bevacizumab, and SBRT + chemotherapy + cetuximab were as follows: 92.0% vs. 96.2% vs. 94.4%, 28.0% vs. 74.7% vs. 63.5%, and 20.0% vs. 50.0% vs. 42.3%, respectively (**Figure 1C**).

### Factors affecting the treatment outcomes

In our comprehensive univariate analysis (**Table 3**), we analyzed the influence of various factors, including age, gender, primary tumor site, tumor size, BED_10_ value, ECOG-PS score, and treatment on OS, PFS, and LCRs. We observed that tumor size <2 cm and BED_10_ ≥ 100 Gy were correlated with improved local control (*p* < 0.05), and other factors had no relevant statistical significance. For BED_10_ ≥ 100 Gy, the primary site was the rectum, and patients under targeted therapy of bevacizumab or cetuximab were associated with improved OS (*p* < 0.05). Moreover, patients under targeted therapy of bevacizumab or cetuximab and ECOG-PS = 0 were linked to prolonged PFS (*p* < 0.05).

**Table 3:** Univariate analysis for overall survival, progression-free survival, and local control.

Factors	Overall survival			Progression-free survival			Local control		
	Hazard ratio	95% CI	*P*	Hazard ratio	95% CI	*P*	Hazard ratio	95% CI	*P*
Age (<60 vs. ≥60)	0.70	0.39–1.25	0.23	0.87	0.53–1.43	0.58	0.94	0.48–1.83	0.86
Gender (female vs. male)	0.81	0.45–1.48	0.50	1.15	0.69–1.90	0.60	1.02	0.52–2.01	0.95
Primary site (rectum vs. colon)	**0.46**	0.25–0.84	**0.01**	0.81	0.47–1.38	0.43	0.69	0.34–1.39	0.29
Treatment (chemotherapy vs. targeted therapy)	**2.66**	1.48–4.76	**0.00**	**3.91**	2.16–7.08	**0.00**	1.99	0.99–3.40	0.05
Tumor size (<2 vs. ≥2)	0.52	0.27–1.02	0.06	0.57	0.31–1.02	0.06	**0.28**	0.14–0.58	**0.00**
BED_10_ (<100 vs. ≥100)	**2.12**	1.05–4.30	**0.04**	1.32	0.71–2.44	0.40	**2.29**	1.05–5.01	**0.04**
ECOG PS	0.52	0.26–1.03	0.06	**0.47**	0.27–0.82	**0.01**	0.55	0.26–1.18	0.13

ECOG, Eastern Cooperative Oncology Group; BED, biologically effective dose.

Bolded HRs and P-values indicate statistical significance (*P* < 0.05).

In our comprehensive multivariate analysis (**Table 4**), we identified that tumor size <2 cm was associated with better local control (*p* < 0.05). The remaining factors analyzed were not statistically relevant. Patients under targeted therapy were linked to prolonged OS (*p* < 0.05). Furthermore, there exhibited significant associations with improved PFS (*p* < 0.05): tumor size <2 cm and patients undergoing targeted therapy.

**Table 4:** Multivariate analysis for overall survival, progression-free survival, and local control.

Factors	Overall survival			Progression-free survival			Local control		
	Hazard ratio	95% CI	*P*	Hazard ratio	95% CI	*P*	Hazard ratio	95% CI	*P*
Age (<60 vs. ≥60)	0.88	0.45–1.72	0.72	0.93	0.51–1.68	0.80	0.85	0.40–1.81	0.67
Gender (female vs. male)	0.89	0.46–1.71	0.73	1.22	0.69–2.14	0.50	1.30	0.58–2.93	0.53
Primary site (rectum vs. colon)	0.63	0.33–1.21	0.16	1.09	0.60–1.98	0.77	1.41	0.61–3.22	0.42
Treatment (chemotherapy vs. targeted therapy)	**2.05**	1.07–3.92	**0.03**	**3.67**	1.96–6.89	**0.00**	1.91	0.90–4.07	0.09
Tumor size (<2 vs. ≥2)	0.70	0.33–1.49	0.35	**0.50**	0.26–0.97	**0.04**	**0.28**	0.12–0.66	**0.00**
BED_10_ (<100 vs. ≥100)	1.46	0.63–3.63	0.38	1.23	0.60–2.53	0.58	1.88	0.71–5.02	0.21
ECOG PS	0.70	0.33–1.50	0.36	0.62	0.33–1.17	0.14	0.75	0.33–1.72	0.50

ECOG, Eastern Cooperative Oncology Group; BED, biologically effective dose.

Bolded HRs and P-values indicate statistical significance (*P* < 0.05).

### Toxicity

No instances of grade 4 or 5 radiation pneumonitis were observed, and there were no other toxicities reported at or above grade 4. Notably, patients in the SBRT + chemotherapy arm exhibited the lowest incidence of AEs.

The incidence rates of grade 2–3 radiation pneumonitis were 8% (2/25) in the SBRT + chemotherapy arm compared to 19.2% (5/26) in the SBRT + chemotherapy + bevacizumab arm. In the SBRT + chemotherapy + cetuximab arm, 16.7% (3/18) patients experienced grade 2–3 radiation pneumonitis.

Patients on targeted therapies exhibited elevated rates of hematologic toxicities (*p* < 0.05). The hematological toxicity rates in the SBRT + chemotherapy arm, SBRT + chemotherapy + bevacizumab arm, and SBRT + chemotherapy + bevacizumab arm were 4% (1/25), 38.5% (10/26), and 38.9% (7/18), respectively. Grade 2 toxicity occurred in one patient in the SBRT + chemotherapy arm. Grade 3 occurred in one patient, grade 2 in six patients, and grade 1 in three patients in the SBRT + chemotherapy + bevacizumab arm. In the SBRT + chemotherapy + cetuximab arm, four patients had grade 1, and two had grade 2 toxicity. Furthermore, three patients treated with SBRT + chemotherapy + cetuximab presented grade 1–2 rash.

## Discussion

Current evidence indicates that SBRT is effective in managing oligometastases from CRC (21, 22). It is recognized that OLTs originating from CRC are connected with decreased PFS (23), and such tumors exhibit lower radiosensitivity than other subtypes, possibly due to increased hypoxia and extensive micro-invasion (24–26). To enhance SBRT’s performance as a treatment option for OLTs from CRCs, we explored the use of radiotherapy sensitizers in a retrospective study. By combining cetuximab or bevacizumab with SBRT-based chemoradiotherapy, we aimed to improve local control and survival outcomes.

We found that the addition of bevacizumab or cetuximab to SBRT-based concurrent chemoradiotherapy led to prolonged OS and PFS and improved local tumor control. In certain patients, local lesions remained stable or even regressed for a duration exceeding 2 years, with LCR at 1 year surpassing 90%. While clinical data for cetuximab or bevacizumab treatment in the context of CRC are limited, our findings align with those of previous studies (14, 27, 28). Cetuximab triggered DNA damage and hampered CRC cell proliferation *in vitro* and reduced tumor growth in mouse models, ultimately increasing the radiosensitivity of CRC cell lines (15, 16). Prior studies have also demonstrated that bevacizumab combined with chemoradiotherapy could improve survival in mCRC: metastatic colorectal cancer cases (29–32). Anti-VEGF therapy exhibited radiosensitizing properties in both tumor cell lines and mouse models (17). This phenomenon may be attributed to radiotherapy, triggering a vascular rebound effect, thereby inducing the growth of new blood vessels. This, in turn, results in tissue hypoxia and promotes the heightened expression of VEGF with downstream effects on cell signaling (33–35). In this study, cetuximab exhibited a shorter PFS compared to bevacizumab. We found that liver metastasis occurred early in 44.44% of patients post-treatment with SBRT + chemotherapy + cetuximab, whereas only 15.38% of patients receiving SBRT + chemotherapy + bevacizumab group experienced liver metastasis after treatment. Our data draw a potential link between bevacizumab treatment, tumor angiogenesis inhibition, and reduced early liver metastasis incidence. Our current findings from a limited sample size warrant future validation through large-scale studies.

In our comprehensive univariate analysis, BED_10_ ≥ 100 Gy was associated with improved OS and better local control. Patients under targeted therapy showed improved OS and PFS, and tumor size <2 cm was linked to better local control. These findings aligned with prior research outcomes, as multiple studies have consistently indicated that a higher BED_10_ was linked to improved local control and enhanced survival in cases of mCRC with lung oligometastases (8, 36, 37). Yu et al. found that BED_10_ ≥ 100 Gy was more effective than BED_10_ < 100 Gy in patients with OM-CRC: oligometastatic colorectal cancer in terms of 1-year local control (94.4% vs. 63.2%, *p* = 0.022) and 1-year OS (100% vs. 73.4%, *p* = 0.028) (37). Alongi et al. also reported an excellent 2-year local PFS rate of 80% for colorectal metastases treated with a median BED_10_ of 105 Gy (38). Another study also confirmed that the 2-year cumulative local treatment failure rate for colorectal metastases treated with BED_10_ < 100 Gy was significantly higher than that for colorectal metastases treated with BED_10_ ≥ 100 Gy at 62.5% and 16.7%, respectively (*p* < 0.08) (39). Researchers found that lung metastases from CRC were radioresistant, resulting in high recurrence rates after SBRT. Hence, to improve local tumor control, they recommend dose escalation with BED_10_ > 100 Gy (36, 40). Our earlier investigation revealed that OLTs treated with higher doses (BED_10_ = 132 Gy) exhibited superior local control rates compared to those treated with lower doses (BED_10_ ≤ 105.6 Gy) (12). In this study, the targeted agent functioned as a sensitizer, enhancing local control even at doses of BED_10_ ≥ 100 Gy. Prior studies have highlighted the significance of tumor size before SBRT as a prognostic factor. Patients with larger lung metastases demonstrated poorer local control (41, 42). Both the univariate and multivariate analyses in this study revealed that, in comparison to patients with tumors ≥2 cm, those with tumor size <2 cm exhibited improved local control. This is consistent with previous findings. For larger tumors with a higher chance of hidden distant spread, a higher radiation dose may be necessary for effective control (43). We also investigated the relationship between the number of lung lesions treated simultaneously and local control rates. However, due to the inconsistency in the systemic treatment regimens among the groups, baseline heterogeneity, and the small sample size, we were unable to draw definitive conclusions from this statistical result. In future studies, we plan to increase the sample size, standardize patient baseline characteristics, and conduct a more in-depth investigation of this issue. Our univariate analysis revealed patients with better performance status had longer PFS, which was consistent with the results of Ji et al. (5).

Here, we found that cetuximab or bevacizumab treatment showed slightly higher rates of hematologic toxicity in this research, consistent with previous studies in CRC and recurrent malignant glioma (18, 44, 45). However, relevant studies in colorectal cancer are currently very limited, and further research with larger sample sizes is needed. The combination of radiotherapy with bevacizumab or cetuximab showed a trend toward increased radiation pneumonitis, although this finding was not statistically significant. Further validation with a larger sample size will substantiate this observation. Moreover, previous studies also reported that bevacizumab treatment could be linked to the inhibition of the repair of damaged lung endothelial tissues and the recruitment of inflammatory factors (46). Also, cetuximab was found to cause rash due to its involvement in releasing pro-inflammatory chemokines and attracting T-cell and neutrophil infiltration (47). Nevertheless, in our current study, we found that the overall toxicity of combination therapy was tolerable.

This single-center retrospective study has several limitations, including the small patient cohort and retrospective design. Due to the retrospective nature of the research, its validity is inherently limited. The study included all eligible patients between March 2011 and March 2023, ensuring comprehensive inclusion to minimize selection bias as much as possible. Many patients were in the advanced stages of CRC, and the majority had undergone multiple lines of treatment before receiving SBRT + chemotherapy, which inevitably affected the efficacy of SBRT. Limited insurance coverage for some patients meant they lacked access to genetic testing and chemotherapy with cetuximab/bevacizumab. Our current findings should be validated in larger, preferably prospective studies. Additionally, screening for predictive biomarkers is recommended when combining anti-angiogenic drugs for more precise treatment.

## Conclusion

This single-site study shows that a combination of SBRT-based concurrent chemoradiotherapy with bevacizumab or cetuximab is effective for treating pulmonary oligometastases from CRC. Retrospective analyses consistently showed improved LCR, PFS, and OS with cetuximab or bevacizumab.

## Data Availability

The original contributions presented in the study are included in the article/supplementary material. Further inquiries can be directed to the corresponding authors.

## References

[1] Sung H Ferlay J Siegel RL Laversanne M Soerjomataram I Jemal A. Global cancer statistics 2020: GLOBOCAN estimates of incidence and mortality worldwide for 36 cancers in 185 countries. CA Cancer J Clin. (2021) 71:209–49. doi: 10.3322/caac.21660 33538338

[2] Shin AE Giancotti FG Rustgi AK. Metastatic colorectal cancer: mechanisms and emerging therapeutics. Trends Pharmacol Sci. (2023) 44:222–36. doi: 10.1016/j.tips.2023.01.003 PMC10365888 36828759

[3] Penna C Nordlinger B. Colorectal metastasis (liver and lung). Surg Clin North Am. (2002) 82:1075–90. doi: 10.1016/s0039-6109(02)00051-8 12507210

[4] Pastorino U Buyse M Friedel G Ginsberg RJ Girard P Goldstraw P. Long-term results of lung metastasectomy: prognostic analyses based on 5206 cases. J Thorac Cardiovasc Surg. (1997) 113:37–49. doi: 10.1016/s0022-5223(97)70397-0 9011700

[5] Ji X Zhao Y Zhu X Shen Z Li A Chen C. Outcomes of stereotactic body radiotherapy for metastatic colorectal cancer with oligometastases, oligoprogression, or local control of dominant tumors. Front Oncol. (2020) 10:595781. doi: 10.3389/fonc.2020.595781 33585211 PMC7878536

[6] Navarria P De Rose F Ascolese AM. SBRT for lung oligometastases: Who is the perfect candidate? Rep Pract Oncol Radiother. (2015) 20:446–53. doi: 10.1016/j.rpor.2014.11.005 PMC4661350 26696785

[7] Simmonds PC. Palliative chemotherapy for advanced colorectal cancer: systematic review and meta-analysis. Colorectal Cancer Collaborative Group. Bmj. (2000) 321:531–5. doi: 10.1136/bmj.321.7260.531 PMC27466 10968812

[8] Kobiela J Spychalski P Marvaso G Ciardo D Dell'Acqua V Kraja F. Ablative stereotactic radiotherapy for oligometastatic colorectal cancer: Systematic review. Crit Rev Oncol Hematol. (2018) 129:91–101. doi: 10.1016/j.critrevonc.2018.06.005 30097241

[9] Takeda A Kunieda E Ohashi T Aoki Y Koike N Takeda T. Stereotactic body radiotherapy (SBRT) for oligometastatic lung tumors from colorectal cancer and other primary cancers in comparison with primary lung cancer. Radiother Oncol. (2011) 101:255–9. doi: 10.1016/j.radonc.2011.05.033 21641064

[10] Norihisa Y Nagata Y Takayama K Matsuo Y Sakamoto T Sakamoto M. Stereotactic body radiotherapy for oligometastatic lung tumors. Int J Radiat Oncol Biol Phys. (2008) 72:398–403. doi: 10.1016/j.ijrobp.2008.01.002 18374506

[11] Jingu K Matsushita H Yamamoto T Umezawa R Ishikawa Y Takahashi N. Stereotactic radiotherapy for pulmonary oligometastases from colorectal cancer: A systematic review and meta-analysis. Technol Cancer Res Treat. (2018) 17:1533033818794936. doi: 10.1177/1533033818794936 30145943 PMC6111389

[12] Wang X Zamdborg L Ye H Grills IS Yan D. A matched-pair analysis of stereotactic body radiotherapy (SBRT) for oligometastatic lung tumors from colorectal cancer versus early stage non-small cell lung cancer. BMC Cancer. (2018) 18:962. doi: 10.1186/s12885-018-4865-9 30305131 PMC6180414

[13] Bonner JA Harari PM Giralt J Azarnia N Shin DM Cohen RB. Radiotherapy plus cetuximab for squamous-cell carcinoma of the head and neck. N Engl J Med. (2006) 354:567–78. doi: 10.1056/NEJMoa053422 16467544

[14] Comet B Kramar A Faivre-Pierret M Dewas S Coche-Dequeant B Degardin M. Salvage stereotactic reirradiation with or without cetuximab for locally recurrent head-and-neck cancer: a feasibility study. Int J Radiat Oncol Biol Phys. (2012) 84:203–9. doi: 10.1016/j.ijrobp.2011.11.054 22331006

[15] Shin HK Kim MS Lee JK Lee SS Ji YH Kim JI. Combination effect of cetuximab with radiation in colorectal cancer cells. Tumori. (2010) 96:713–20. doi: 10.1177/030089161009600513 21302618

[16] Vassileva V Rajkumar V Mazzantini M Robson M Badar A Sharma S. Significant therapeutic efficacy with combined radioimmunotherapy and cetuximab in preclinical models of colorectal cancer. J Nucl Med. (2015) 56:1239–45. doi: 10.2967/jnumed.115.157362 26045312

[17] Gorski DH Beckett MA Jaskowiak NT Calvin DP Mauceri HJ Salloum RM. Blockage of the vascular endothelial growth factor stress response increases the antitumor effects of ionizing radiation. Cancer Res. (1999) 59:3374–8. 10416597

[18] Mazzola R Tebano U Aiello D Paola GD Giaj-Levra N Ricchetti F. Increased efficacy of stereotactic ablative radiation therapy after bevacizumab in lung oligometastases from colon cancer. Tumori. (2018) 104:423–8. doi: 10.5301/tj.5000701 29737958

[19] Hindocha S Campbell D Ahmed M Giorgakoudi K Sharma B Yousaf N. Immune checkpoint inhibitor and radiotherapy-related pneumonitis: an informatics approach to determine real-world incidence, severity, management, and resource implications. Front Med (Lausanne). (2021) 8:764563. doi: 10.3389/fmed.2021.764563 34790682 PMC8591134

[20] Yan Y Fu J Kowalchuk RO Wright CM Zhang R Li X. Exploration of radiation-induced lung injury, from mechanism to treatment: a narrative review. Transl Lung Cancer Res. (2022) 11:307–22. doi: 10.21037/tlcr-22-108 PMC8902083 35280316

[21] Agolli L Bracci S Nicosia L Valeriani M De Sanctis V Osti MF. Lung metastases treated with stereotactic ablative radiation therapy in oligometastatic colorectal cancer patients: outcomes and prognostic factors after long-term follow-up. Clin Colorectal Cancer. (2017) 16:58–64. doi: 10.1016/j.clcc.2016.07.004 27522627

[22] Carconi C Cerreti M Roberto M Arrivi G D'Ambrosio G De Felice F. The management of oligometastatic disease in colorectal cancer: Present strategies and future perspectives. Crit Rev Oncol Hematol. (2023) 186:103990. doi: 10.1016/j.critrevonc.2023.103990 37061075

[23] Osti MF Agolli L Valeriani M Reverberi C Bracci S Marinelli L. 30 Gy single dose stereotactic body radiation therapy (SBRT): Report on outcome in a large series of patients with lung oligometastatic disease. Lung Cancer. (2018) 122:165–70. doi: 10.1016/j.lungcan.2018.06.018 30032826

[24] Mendez Romero A Seppenwoolde Y Verheij J Dwarkasing RS Verhoef C Redekop WK. Macroscopic and microscopic pathologic findings of colorectal liver metastases correlated with magnetic resonance imaging to establish safety margins for stereotactic body radiation therapy. Int J Radiat Oncology Biology Physics. (2010) 78:S56. doi: 10.1016/j.ijrobp.2010.07.164

[25] Goethals L Debucquoy A Perneel C Geboes K Ectors N De Schutter H. Hypoxia in human colorectal adenocarcinoma: comparison between extrinsic and potential intrinsic hypoxia markers. Int J Radiat Oncol Biol Phys. (2006) 65:246–54. doi: 10.1016/j.ijrobp.2006.01.007 16618579

[26] van Laarhoven HW Kaanders JH Lok J Peeters WJ Rijken PF Wiering B. Hypoxia in relation to vasculature and proliferation in liver metastases in patients with colorectal cancer. Int J Radiat Oncol Biol Phys. (2006) 64:473–82. doi: 10.1016/j.ijrobp.2005.07.982 16242253

[27] Robert F Ezekiel MP Spencer SA Meredith RF Bonner JA Khazaeli MB. Phase I study of anti–epidermal growth factor receptor antibody cetuximab in combination with radiation therapy in patients with advanced head and neck cancer. J Clin Oncol. (2001) 19:3234–43. doi: 10.1200/jco.2001.19.13.3234 11432891

[28] Bonner JA Harari PM Giralt J Cohen RB Jones CU Sur RK. Radiotherapy plus cetuximab for locoregionally advanced head and neck cancer: 5-year survival data from a phase 3 randomised trial, and relation between cetuximab-induced rash and survival. Lancet Oncol. (2010) 11:21–8. doi: 10.1016/s1470-2045(09)70311-0 19897418

[29] Hurwitz H Fehrenbacher L Novotny W Cartwright T Hainsworth J Heim W. Bevacizumab plus irinotecan, fluorouracil, and leucovorin for metastatic colorectal cancer. N Engl J Med. (2004) 350:2335–42. doi: 10.1056/NEJMoa032691 15175435

[30] Crane CH Eng C Feig BW Das P Skibber JM Chang GJ. Phase II trial of neoadjuvant bevacizumab, capecitabine, and radiotherapy for locally advanced rectal cancer. Int J Radiat Oncol Biol Phys. (2010) 76:824–30. doi: 10.1016/j.ijrobp.2009.02.037 19464823

[31] Kennecke H Berry S Wong R Zhou C Tankel K Easaw J. Pre-operative bevacizumab, capecitabine, oxaliplatin and radiation among patients with locally advanced or low rectal cancer: a phase II trial. Eur J Cancer. (2012) 48:37–45. doi: 10.1016/j.ejca.2011.05.016 21664123

[32] Willett CG Duda DG di Tomaso E Boucher Y Ancukiewicz M Sahani DV. Efficacy, safety, and biomarkers of neoadjuvant bevacizumab, radiation therapy, and fluorouracil in rectal cancer: a multidisciplinary phase II study. J Clin Oncol. (2009) 27:3020–6. doi: 10.1200/JCO.2008.21.1771 PMC2702234 19470921

[33] Wang K Chen Y Zhang Z Wu R Zhou M Yang W. RIFLE: a Phase II trial of stereotactic ablative radiotherapy combined with fruquintinib and tislelizumab in metastatic colorectal cancer. Gastroenterol Rep (Oxf). (2023) 11:goad063. doi: 10.1093/gastro/goad063 37842200 PMC10568524

[34] Goedegebuure RSA de Klerk LK Bass AJ Derks S Thijssen V. Combining radiotherapy with anti-angiogenic therapy and immunotherapy; A therapeutic triad for cancer? Front Immunol. (2018) 9:3107. doi: 10.3389/fimmu.2018.03107 30692993 PMC6339950

[35] Loncaster JA Cooper RA Logue JP Davidson SE Hunter RD West CM. Vascular endothelial growth factor (VEGF) expression is a prognostic factor for radiotherapy outcome in advanced carcinoma of the cervix. Br J Cancer. (2000) 83:620–5. doi: 10.1054/bjoc.2000.1319 PMC2363503 10944602

[36] Ahmed KA Scott JG Arrington JA Naghavi AO Grass GD Perez BA. Radiosensitivity of lung metastases by primary histology and implications for stereotactic body radiation therapy using the genomically adjusted radiation dose. J Thorac Oncol. (2018) 13:1121–7. doi: 10.1016/j.jtho.2018.04.027 PMC7810135 29733909

[37] Yu J Li N Tang Y Wang X Tang Y Wang SL. Outcomes after hypofractionated stereotactic radiotherapy for colorectal cancer oligometastases. J Surg Oncol. (2019) 119:532–8. doi: 10.1002/jso.25361 30609038

[38] Nicosia L Cuccia F Mazzola R Ricchetti F Figlia V Giaj-Levra N. Disease course of lung oligometastatic colorectal cancer treated with stereotactic body radiotherapy. Strahlenther Onkol. (2020) 196:813–20. doi: 10.1007/s00066-020-01627-7 32399637

[39] Binkley MS Trakul N Jacobs LR von Eyben R Le QT Maxim PG. Colorectal histology is associated with an increased risk of local failure in lung metastases treated with stereotactic ablative radiation therapy. Int J Radiat Oncol Biol Phys. (2015) 92:1044–52. doi: 10.1016/j.ijrobp.2015.04.004 26025776

[40] Ricco A Davis J Rate W Yang J Perry D Pablo J. Lung metastases treated with stereotactic body radiotherapy: the RSSearch® patient Registry's experience. Radiat Oncol. (2017) 12:35. doi: 10.1186/s13014-017-0773-4 28143558 PMC5286804

[41] Kobayashi N Abe T Noda SE Kumazaki YU Hirai R Igari M. Stereotactic body radiotherapy for pulmonary oligometastasis from colorectal cancer. In Vivo. (2020) 34:2991–6. doi: 10.21873/invivo.12130 PMC7652475 32871842

[42] Jung J Song SY Kim JH Yu CS Kim JC Kim TW. Clinical efficacy of stereotactic ablative radiotherapy for lung metastases arising from colorectal cancer. Radiat Oncol. (2015) 10:238. doi: 10.1186/s13014-015-0546-x 26588896 PMC4654895

[43] Nagata Y Takayama K Matsuo Y Norihisa Y Mizowaki T Sakamoto T. Clinical outcomes of a phase I/II study of 48 Gy of stereotactic body radiotherapy in 4 fractions for primary lung cancer using a stereotactic body frame. Int J Radiat Oncol Biol Phys. (2005) 63:1427–31. doi: 10.1016/j.ijrobp.2005.05.034 16169670

[44] Cabrera AR Cuneo KC Vredenburgh JJ Sampson JH Kirkpatrick JP. Stereotactic radiosurgery and bevacizumab for recurrent glioblastoma multiforme. J Natl Compr Canc Netw. (2012) 10:695–9. doi: 10.6004/jnccn.2012.0072 22679114

[45] Gutin PH Iwamoto FM Beal K Mohile NA Karimi S Hou BL. Safety and efficacy of bevacizumab with hypofractionated stereotactic irradiation for recurrent Malignant gliomas. Int J Radiat Oncol Biol Phys. (2009) 75:156–63. doi: 10.1016/j.ijrobp.2008.10.043 PMC3659401 19167838

[46] Chen F Niu J Wang M Zhu H Guo Z. Re-evaluating the risk factors for radiation pneumonitis in the era of immunotherapy. J Transl Med. (2023) 21:368. doi: 10.1186/s12967-023-04212-5 37287014 PMC10246421

[47] Tougeron D Emambux S Favot L Lecomte T Wierzbicka-Hainaut E Samimi M. Skin in-flammatory response and efficacy of anti-epidermal growth factor receptor therapy in metastatic colorectal cancer (CUTACETUX). Oncoimmunology. (2020) 9:1848058. doi: 10.1080/2162402x.2020.1848058 33299659 PMC7714491

